# Carmustine-Induced Takotsubo Cardiomyopathy

**DOI:** 10.7759/cureus.51122

**Published:** 2023-12-26

**Authors:** Muhammad Z Khan, Muhammad Waqas, Hadia Shah, Sona Franklin, Ayesha Jamil

**Affiliations:** 1 Cardiology, Thomas Jefferson University Hospital, Philadelphia, USA; 2 Internal Medicine, Swat Medical College, Saidu Sharif, PAK; 3 Internal Medicine, Saidu Medical College, Saidu Sharif, PAK; 4 Internal Medicine, St Mary Medical Center, Langhorne, USA; 5 Medicine, St Mary Medical Center, Langhorne, USA

**Keywords:** drug-induced takosubo cardiomyopathy, heart failure, stress induced cardiomyopathy, carmustine, takotsubo cardiomyopathy (ttc)

## Abstract

Carmustine is an intravenous alkylating agent that inhibits DNA synthesis and protein synthesis by forming cross-links in DNA and RNA and ultimately causing cell death. We report a rare case of Takotsubo cardiomyopathy that is thought to be related to carmustine, where the patient presented with chest pain, and ST depression within 12 hours of carmustine therapy. Workup, including echocardiography, showed global hypokinesis of the left ventricle with regional variations; mid and apical anterior septum and apex were akinetic with left ventricular ejection fraction (EF) of 30%. Cardiac catheterization showed nonobstructive coronary artery disease. The patient was treated with a beta-blocker, angiotensin receptor-neprilysin inhibitor (ARNi), and aldosterone receptor antagonists. Two days later, he had a repeat echocardiogram that showed improved EF. After stem cell infusion, his course was complicated with atrial fibrillation with rapid ventricular response and septic shock. Unfortunately, he suffered a cardiac arrest and expired. Carmustine-related cardiomyopathy seems to be very rare, and, to our knowledge, this is the first case report.

## Introduction

Takotsubo cardiomyopathy (TCM) is defined as transient regional systolic dysfunction in the absence of obstructive coronary artery disease, typically accompanied by ST-segment changes/T-wave inversions with raised troponin levels [1,2].

Chemotherapeutic drugs have been linked to several heart disorders, including TCM. The pathophysiology of chemotherapy-induced TCM remains uncertain. It is a hypothesis that it is likely due to emotional and physiological stress, paraneoplastic phenomena, and direct cardiotoxicity caused by free radicals [2]. Carmustine is a nonspecific alkylating antineoplastic agent used for brain cancer (including glioma, glioblastoma multiforme, medulloblastoma, and astrocytoma), multiple myeloma, and lymphoma (Hodgkin's and non-Hodgkin's) [3]. Carmustine-induced cardiotoxicity is rare but may cause chest pain, hypotension, and sinus tachycardia [3]. However, there is no published literature on carmustine-induced TCM. Here we report a case in which a patient developed reversible cardiomyopathy after carmustine induction. 

## Case presentation

**Figure 1:**
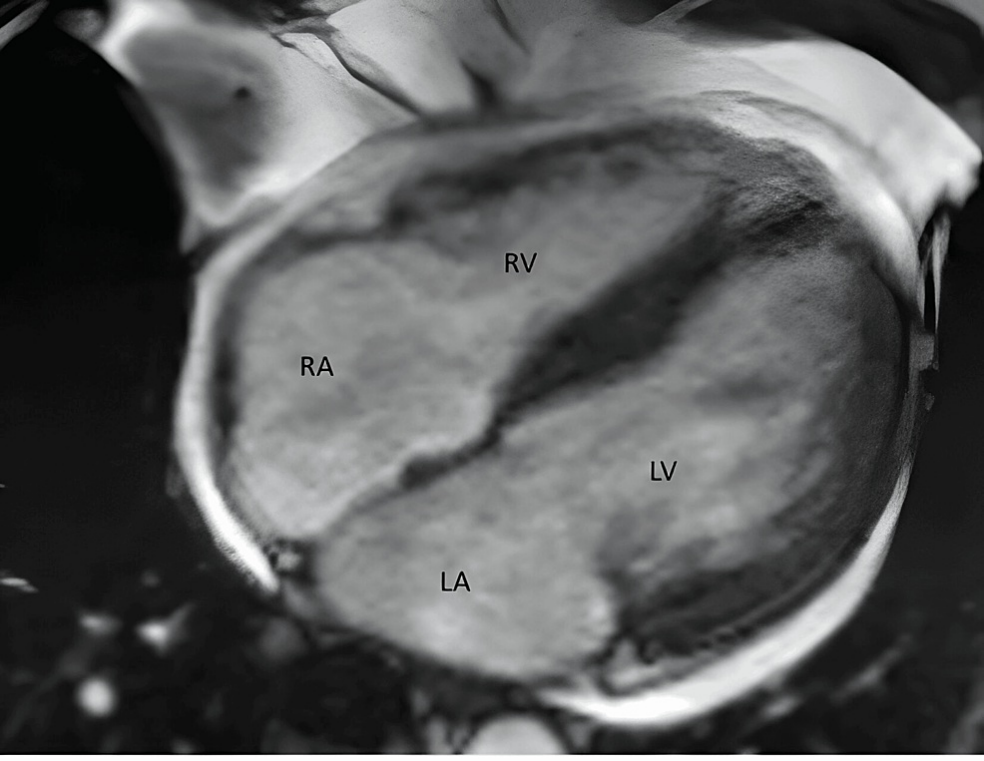
Cardiac magnetic resonance imaging, demonstrating normal systolic function RA, right atrium; LA, left atrium; LV, left ventricle; RV, right ventricle

A 75-year-old man was admitted for autologous stem cell transplant with a carmustine-containing conditioning regimen. His past medical history was notable for hypertension, chronic obstructive pulmonary disease, hyperlipidemia, atrial fibrillation, and peripheral T-cell lymphoma with T-follicular helper (TFH) phenotype, that was treated with 6 cycles of CEOP (cyclophosphamide, etoposide, vincristine, and prednisone) with the achievement of complete remission. Five weeks before receiving carmustine, his cardiac MRI showed normal left ventricle (LV) size and systolic function (left ventricular ejection fraction or LVEF, 54%) with no evidence of regional wall motion abnormalities and no myocardial inflammation/edema on late gadolinium imaging (Figure 1).

**Figure 2:**
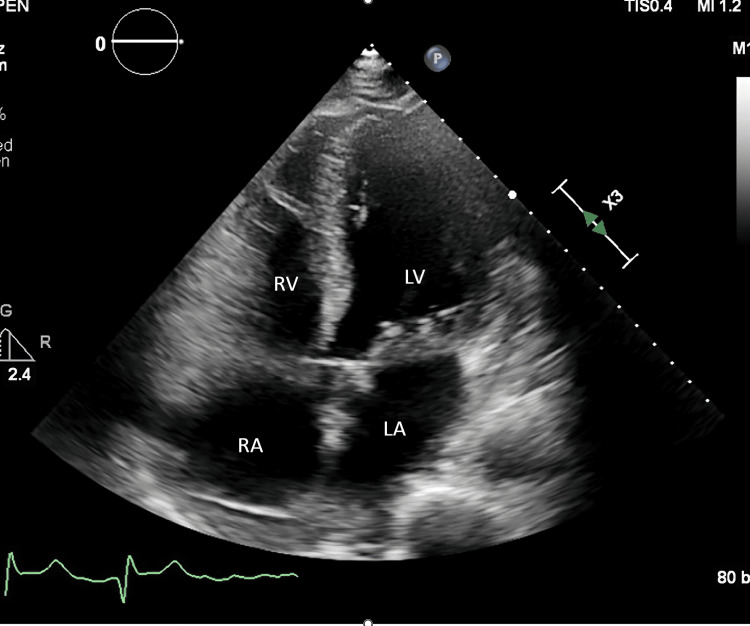
Echocardiogram of the patient showing akinetic apex and global hypokinesis of the left ventricle with regional variations RA, right atrium; LA, left atrium; LV, left ventricle; RV, right ventricle

He received carmustine 300 mg/m2 on the first day of the conditioning regimen and developed chest pain 12 hours after the infusion, an EKG was done, which showed ST depressions in lateral leads. Troponin was 0.13 ng/ml, 0.12 ng/ml, 0.14 ng/ml (normal troponin< 0.03 ng/ml). Echocardiography was done, which showed EF of 30% global hypokinesis of the left ventricle with regional variations; mid and apical anterior septum and apex were akinetic (Figure 2).

**Figure 3:**
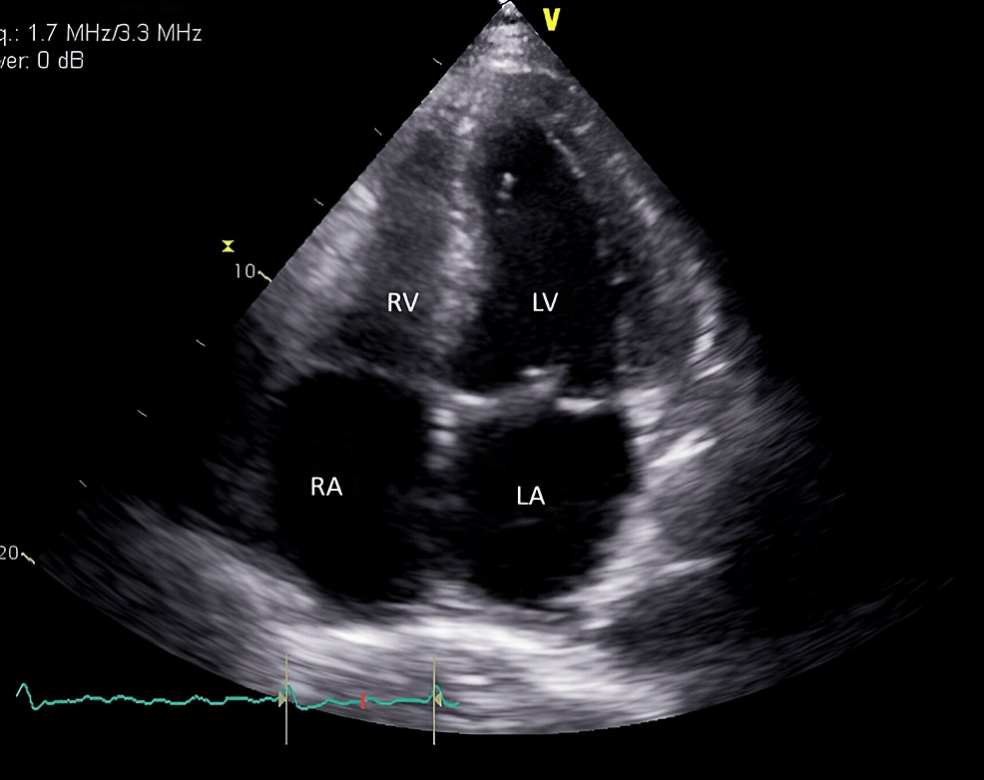
Echocardiogram of the patient showing improved regional wall motions RA, right atrium; LA, left atrium; LV, left ventricle; RV, right ventricle

A left heart catheterization showed mild non-obstructive coronary artery disease in the right dominant circulation. Sacubitril/valsartan 24 mg/26 mg bid, empagliflozin 5 mg daily, spironolactone 12.5 mg daily, and isosorbide mononitrate 30 mg daily were initiated after establishing diagnoses of TCM. An echocardiogram was repeated after two days, which showed improved regional wall motions, LVEF 40% (Figure 3).

The decision was made to proceed with the remaining conditioning regimen, which included etoposide, cytarabine, and melphalan. Carmustin was discontinued. Stem cell infusion was performed on the day following the completion of the conditioning regimen. Per protocol, filgrastim 480 mcg daily was initiated five days after stem cell infusion. Acyclovir and fluconazole started prophylactically. Two days later after stem cell infusion, the patient went into atrial fibrillation (AF) with rapid ventricular response (RVR), which was initially treated with metoprolol and then IV amiodarone. Chest X-ray was a concern for perihilar interstitial prominence suggestive of pulmonary edema and hazy bibasilar opacities suggestive of consolidation/atelectasis. The patient became hypotensive, and he was febrile. They started on norepinephrine, epinephrine, and vasopressin. The patient was treated with vancomycin/cefepime, micafungin, and doxycycline for neutropenic fever. An infectious workup with blood culture, urine culture, urine legionella, and strep was negative. Unfortunately, the patient suffered a cardiac arrest (asystole) and expired. 

## Discussion

TCM, also known as stress cardiomyopathy, most commonly occurs in postmenopausal females [1]. Only 2% of the patients who present to the hospital with acute coronary syndrome are diagnosed with TCM [2]. Twenty-seven cases of chemotherapy-related TCM have been reported in the literature since 2000, out of which 19 were attributed to chemotherapy, 6 to monoclonal antibodies and 2 to tyrosine kinase inhibitor therapies [3]. Here we present the first case of TCM secondary to the alkylating agent, carmustine.

The clinical presentation of TCM is like acute coronary syndrome with acute retrosternal chest pain being the most common symptom. Other signs and symptoms may include dyspnea, syncope, arrhythmias, or sudden cardiac arrest. It is a transient condition, and treatment is usually conservative, focusing on treating the underlying cause [4]. The prognosis is usually favorable with complete recovery in a few weeks.

Our patient developed chest pain 12 hours after receiving carmustine which is an alkylating agent that inhibits DNA/RNA synthesis and protein synthesis by forming cross-links in DNA and RNA and ultimately causing cell death and is usually given by an intravenous route [5]. The exact mechanism of cardiotoxicity caused by carmustine is not known. However, the general mechanism involved behind chemotherapy-induced TCM is increased sympathetic tone and increased production of cytokines, free radicals, prostaglandins, catecholamine, and growth factor levels which worsens adrenoreceptor sensitivity [4]. These chemical mediators damage cell membranes by causing lipid peroxidation and activation of pro-apoptotic enzymes. Cardiac myocytes are more predisposed to lipid peroxidation because of the increased number of mitochondria and high energy requirements and decreased production of antioxidant enzymes required to combat free radicals [4]. The most widely accepted theory is that stress causes catecholamine release, which causes microvascular dysfunction or direct cardiac toxicity, eventually leading to myocardial stunning [5].

This case report shows a causal relationship between carmustine and TCM as our patient developed signs and symptoms of TCM approximately twelve hours after he received carmustine. The diagnosis of TCM was confirmed by echocardiography and left heart catheterization. There are few case reports on various chemotherapeutic agents causing TCM, but this is the first case report to show carmustine as the culprit of TCM.

## Conclusions

Patients can develop stress-induced cardiomyopathy after carmustine induction. We would suggest that physicians should be aware of the possible association of carmustine with Takotsubo cardiomyopathy and should always consider an appropriate workup in patients presenting with cardiac symptoms after receiving carmustine.
